# Emerging concepts and recent advances in basal cell carcinoma

**DOI:** 10.12688/f1000research.11314.1

**Published:** 2017-12-04

**Authors:** Mariam Totonchy, David Leffell

**Affiliations:** 1 Department of Dermatology, Yale University School of Medicine, 333 Cedar Street, P.O. Box 208059, New Haven, CT 06520-8059, Connecticut, USA; 2 Department of Dermatology, Section of Cutaneous Oncology and Dermatologic Surgery , Yale University School of Medicine, 40 Temple Street 5A, New Haven, Connecticut, USA

**Keywords:** basal cell carcinoma, non-melanoma, squamous cell, vismodegib

## Abstract

Basal cell carcinoma (BCC) is the most common malignancy worldwide, arising from non-keratinizing cells within the basal layer of the epidermis. The incidence of BCC continues to rise annually, increasing the burden of management of these carcinomas and the morbidity associated with their treatment. While surgical interventions such as Mohs micrographic surgery and surgical excision are the standard of care and yield the highest cure rates, the number of non-surgical interventions approved for the treatment of BCC continues to expand. We review various surgical and non-surgical approaches to the treatment of BCC, focusing on targeted molecular therapies that are approved for locally advanced or recurrent disease.

## Introduction

Non-melanoma skin cancers (NMSCs), which include basal cell carcinoma (BCC) and squamous cell carcinoma (SCC), are the most common cancers in Caucasians. One in five Americans will develop skin cancer in their lifetime ^1^. In 2012, 5.4 million NMSCs were diagnosed in the US alone ^2^. BCC comprises approximately 80% of these NMSCs, and incidence rates are increasing worldwide ^2, 3^. Incidence rates of BCC in the US have risen by approximately 2% per year ^3^, and there are significant increases among women and individuals younger than 40 years of age ^3– 6^. BCCs are treatable cancers and have low rates of metastasis and mortality ^7, 8^; however, their high incidence rates and treatment costs contribute significantly to the rising economic burden of health care ^9^. From 2007 to 2011, as compared with from 2002 to 2006, the aggregate cost of treating skin cancer in the US rose from $3.6 to $8.1 billion per year, and the average cost of treatment per patient has also increased significantly, from $1,000 in 2006 to $1,600 in 2011 ^9– 11^. In addition to the rising cost of treatment, BCCs are associated with significant morbidity, making their management an important public health concern.

## Risk factors

The increase in incidence rates of BCC may indicate changes in environmental or lifestyle and behavioral risk factors. Solar ultraviolet (UV) irradiation is a known risk factor for BCC ^12^. Exposure to UV light, particularly UVB, induces mutations in tumor suppressor genes and plays a key role in the pathogenesis of BCC ^13– 15^. A history of blistering sunburn and younger age at first blistering sunburn have been associated with BCC, yet it is still unclear whether continuous or intermittent sun exposure poses a greater risk ^16– 18^. Exposure to UV radiation early in life or intermittent exposure throughout life may be more important than overall cumulative exposure ^19^. In the past decade, indoor tanning has emerged as a significant risk factor for skin cancer, including early-onset BCC ^20– 24^. The melanocortin 1 receptor gene, *MC1R*, is associated with fair skin, red hair, and increased risk for melanoma and NMSC ^25– 30^. Both MC1R genotype and indoor tanning have been used to predict risk of early-onset BCC in statistical models ^28^.

Increasing age, male sex, and white race have also been correlated with higher rates of BCC ^31, 32^. Higher risk with age may be due in part to a reduced ability to repair DNA damage from UV radiation, leading to the accumulation of carcinogenic photoproducts ^33, 34^. Other important risk factors include a family history of skin cancer, fair complexion, light eye/hair color, and a low ability to tan ^35, 36^. Interestingly, a recent study also found that adult body mass index was inversely associated with early-onset BCC ^37^.

In addition to risk factors for developing BCC, there are certain features of BCCs that place them in a high-risk category. High-risk BCC features include tumors of longstanding duration, diameter of greater than 2 cm, aggressive histo-pathologic subtype, perivascular or perineural infiltration, location in the mid-face or ears, history of radiation exposure, and prior treatment failure ^38^. Morpheaform, infiltrative, and micronodular histo-pathologic subtypes are considered aggressive.

Chronically immunosuppressed patients are at high risk not only for NMSC but also for more aggressive phenotypes of these cutaneous carcinomas due to impaired immune surveillance of the skin ^39– 41^. Solid organ transplant recipients comprise an important subgroup of immunosuppressed patients for whom skin cancer is the most common post-transplant malignancy ^42– 46^. While the relative increased risk of developing SCC is higher compared with BCC in this group, the risk of BCC is still significantly increased at a factor of 7 to 20 as compared with the normal population ^43, 47^. The number of solid organ transplants in the US is increasing annually, making treatment of NMSC in this high-risk group of heightened concern ^48^.

## Genetic basis of disease

UV radiation plays a critical role in the pathogenesis of BCC, accounting for the high rate of somatic mutations and “UV signatures” seen in this type of carcinoma ^49, 50^. In addition to these “UV signature” genetic alterations, mutations in the patched 1 ( *PTCH1*) gene on chromosome 9q22 have been shown to play a key role in the pathogenesis of sporadic BCC ^51, 52^. Loss of heterozygosity of *PTCH1* is the most frequent genetic alteration in BCC, occurring in a reported 53% to 69% of BCCs ^53– 57^. *PTCH* allele alterations are seen in approximately 83% of BCCs ^55^. Critical to this discovery was the characterization of a subset of patients with nevoid BCC syndrome (Gorlin syndrome). This syndrome, first characterized by Gorlin ^58^, is an autosomal dominant disorder caused by mutations in the *PTCH1* gene, resulting in the inappropriate activation of hedgehog (HH) signaling and an increased risk of BCC and developmental defects ^58– 61^. *PTCH1* encodes a protein that functions as the receptor for sonic HH ligands which, when mutated, leads to uncontrolled cell growth and BCC tumorigenesis ^60, 62^. The HH signaling pathway has been the genetic basis for the development of targeted therapies for BCC, which will be discussed below.

Though less critical than *PTCH1* in the pathogenesis of BCC, p53 is another important tumor suppressor gene that has been implicated in BCC tumorigenesis ^54^. P53 plays a critical role in DNA repair and cell cycle regulation ^63^ and has altered expression in a range of tumor types. Reported rates of p53 mutation are between 44% and 56% in BCC ^54, 64, 65^.

## Management

### Targeted molecular therapies

***Smoothened inhibitors.*** Several molecular therapies for BCC have focused on the HH signaling pathway. Binding of HH ligands to *PTCH1* leads to a loss of inhibition of this pathway, leading to activation of smoothened (SMO), a seven-transmembrane protein downstream of *PTCH1*. SMO then interacts with several proteins and ultimately leads to the expression of the GLI family of transcription factors that promote proliferation, survival, and differentiation, key genes involved in BCC tumorigenesis ^59, 66^. Several of the small-molecule targeted therapies therefore have focused on SMO inhibition.

***Vismodegib.*** In 2012, vismodegib became the first SMO inhibitor to gain US Food and Drug Administration (FDA) approval. It was approved for use in metastatic BCC (a very rare occurrence), for locally advanced BCC recurrent after surgery, and for patients who are not candidates for surgical resection or radiation. The initial phase 1 trial studied 33 patients with locally advanced BCC treated with vismodegib at one of three doses (150, 270, or 540 mg daily) and found that 18 out of 33 patients responded ^67^. Two patients had a complete response, defined as a 100% regression of the visible/palpable lesions, and six had a partial response, defined as a more than 50% reduction in tumor diameter. The median duration of response was 12.8 months ^67^. Of the patients who did not respond, 15 had stable disease and four had progression of their BCCs while on treatment ^67^. Interestingly, two patients with progressive disease had elevated GLI1 mRNA levels in tissue samples, raising the question of possible resistance mechanisms in these patients.

The phase II trial of the efficacy and safety of vismodegib in advanced basal-cell carcinoma (ERIVANCE) by Sekulic *et al*. led to the approval of vismodegib ^68^. This trial enrolled 104 patients (33 patients with metastatic BCC and 71 patients with locally advanced BCC) who took 150 mg daily of vismodegib ^68^. In patients with metastatic BCC, the response rate was 30%, whereas in 63 patients with locally advanced BCC, the response rate was 43%. Response rate was defined as a decrease of 30% or more in the externally visible or radiographic dimension or as complete resolution of ulceration (if present at baseline). The median duration of response was 7.6 months ^68^ and the median progression-free survival was 9.5 months according to independent review. All patients had at least one adverse event, and in 12% of patients, adverse events led to discontinuation of vismodegib. The most common adverse events reported with vismodegib include alopecia, dysgeusia, muscle spasms, weight loss, fatigue, decreased appetite, nausea, and diarrhea ^67– 69^.

Similar response rates were seen in a subsequent open-label, two-cohort, multicenter study of 119 patients with advanced BCC who were poor candidates for surgical resection or radiotherapy. They were also treated with vismodegib 150 mg daily. Response rates were 30.8% for those with metastatic disease and 46.4% for those with locally advanced BCC ^70^. Of the 115 patients included as efficacy-evaluable patients, about half (49.5%) experienced stable disease—27 out of 56 patients with locally advanced BCC and 20 out of 39 patients with metastatic BCC. The median treatment period was 5.5 months, and the most common adverse events reported were muscle spasms, dysgeusia, alopecia, and diarrhea ^70^.

The study of vismodegib in patients with advanced basal cell carcinoma (STEVIE) is the largest vismodegib trial to date; 499 patients (468 with locally advanced BCC and 31 with metastatic disease) ^71^. Objective responses were seen in 66.7% of patients with locally advanced BCC and 37.9% of those with metastatic disease. The median time to response for both groups was 2.7 months, and the median duration of the response was 22.7 months. The median duration of vismodegib treatment was 36.4 weeks, and 80% of patients discontinued therapy (36% due to adverse events, 14% due to progressive disease, and 10% from patient request to stop treatment).

Vismodegib has recently been evaluated as a neoadjuvant to surgery for high-risk BCC ^72^. An open-label single-arm study of 15 enrolled patients examined vismodegib 150 mg/day as neoadjuvant for a goal of 3 to 6 months of therapy prior to surgical resection ^72^. A total of 11 out of 15 patients completed the trial. Of the four who could not complete the trial, one was lost to follow-up and two withdrew due to vismodegib-related side effects and one due to an unrelated adverse event. Of the 13 BCCs that were selected for surgery, vismodegib reduced the surgical defect area by 27% from baseline. In the non-recurrent tumors (9 out of 13), vismodegib reduced the surgical defect area by 36%; however, for the recurrent BCCs (4 out of 13), there was no reduction in surgical defect area, introducing the possibility that these tumors had acquired mutations that rendered them resistant to the effects of vismodegib.

Owing to the genetic basis of nevoid basal cell nevus syndrome, patients with this disorder are of particular interest for treatment with SMO inhibitors. A randomized, double-blind, placebo-controlled trial by Tang *et al*. examined treatment with vismodegib in 41 patients with nevoid basal cell nevus syndrome ^73^. After 3 months of therapy, the number of new surgically eligible BCCs was significantly reduced in the vismodegib-treated group as compared with the placebo group (mean of 2 versus 29, respectively, of new surgically eligible BCCs per year) ^73^. Unfortunately, 54% of patients (14 out of 26) receiving vismodegib discontinued treatment because of adverse drug side effects. Overall, while the results of vismodegib treatment have been promising, there remain important questions regarding durability of the response, long-term tolerability of the adverse effects, and acquisition of resistant mutations over time.

***Sonidegib.*** Sonidegib is an SMO inhibitor that gained FDA approval in 2015 for treatment of patients with locally advanced BCC who are not candidates for curative surgery or radiation ^74^. Approval was based on data from the phase II randomized, double-blind Basal Cell Carcinoma Outcomes with LDE225 Treatment (BOLT) trial assessing the efficacy of sonidegib for metastatic or locally advanced BCC ^75^. A total of 230 patients were enrolled and placed on either 200 or 800 mg daily, and objective response rates were 36% (20 out of 55) and 34% (39 out of 116), respectively. There were both greater response rates and fewer adverse events in the 200mg dose sonidegib group as compared with the 800 mg group ^75^. In analyzing objective response rates for tumor types, 43% and 38% of patients with locally advanced BCC and 15% and 17% of patients with metastatic BCC responded in the 200 and 800 mg dosing schedules, respectively. The median time to tumor response was 4 months, and median progression-free survival was 22.1 months ^75– 78^. The adverse effect profile of sonidegib is similar to that of vismodegib.

Although there are no direct head-to-head comparisons of vismodegib with sonidegib, objective response rates were similar using BCC modified Response Criteria In Solid Tumors (BCC-mRECIST), at 48% in the ERIVANCE (vismodegib, 150 mg daily) study with a minimum follow-up of 21 months, and 56% in the BOLT (sonidegib, 200 mg daily) trial with a minimum follow-up of 18 months ^68, 69, 78^. It should be noted that this is an indirect comparison that should be interpreted with caution. Further randomized, double-blind controlled trials comparing both the safety and efficacy of both SMO inhibitors are needed.

***Hedgehog pathway resistance.*** Primary and secondary drug resistances to SMO inhibitors have been reported. Novel heterozygous missense SMO mutations were sequenced in recurrent and resistant BCC tissue to vismodegib ^79, 80^. In cases of secondary resistance, the SMO mutations that were isolated were not present in the primary tumors that had originally responded to treatment, and distinct recurrent nodules of BCC demonstrated unique SMO mutations, suggesting a heterogeneous and dynamic mechanism of resistance that can rapidly arise in recurrent tumor tissue ^79^. In cases of primary resistance in the tumor tissue of origin, given the cost of treatment with SMO inhibitors as compared with genetic sequencing, it may be more cost-effective for all patients to undergo screening prior to the initiation of targeted therapy. Interestingly, a study of nine patients with advanced BCC resistant to treatment with vismodegib also demonstrated resistance to sonidegib, suggesting that chemoresistance can occur between different SMO inhibitors ^81^.

It is appropriate to consider cost in deciding therapeutic options. According to Centers for Medicare and Medicaid Services (CMS) data from 1992 to 1995, the cost per episode of care for NMSC was estimated at $492 in the outpatient setting ^82^. In a more recent study comparing the economic cost of treating patients with advanced versus non-advanced BCC from 2010 to 2014, the mean cost of treating an advanced BCC was $11,143 compared with $1,171 for a non-advanced BCC ^83^. The majority of patients with advanced BCC (93.7%, n = 794) received radiation therapy, at a mean cost of $10,317. Of the 847 patients with advanced BCCs, only three received vismodegib. The cost of vismodegib is $250 per capsule or $7,500 per month ^83^. Treatment length varies by patient, and the treatment is not curative per se, but rather suppressive. However, an expected 10-month treatment course of vismodegib costs approximately $75,000 ^84^. It is important to reserve this treatment approach for patients who are not candidates for surgery or radiation.

### Mohs, surgical excision, and electrodessication and curettage

Standard surgical excision with 4-mm margins is the recommended treatment for BCCs with non-aggressive histology, size of less than 2 cm, and occurrence on low-risk sites where tissue sparing is not critical (trunk and extremities) ^85^. With 4-mm surgical margins, 95% of cases of BCC less than 2 cm were cleared with standard excision ^86^. BCC of the face demonstrates high rates of incomplete excision, and greater efficacy has been demonstrated using Mohs micrographic surgery (MMS) as compared with standard excision ^87– 92^. Five-year rates of recurrence following MMS for primary BCC are approximately 1.4% to 3.2% for primary and 2.4% to 6.7% for recurrent BCCs ^89– 91^. Low rates of recurrence in MMS are attributed to optimal margin control given full histologic examination at the time of surgery of all peripheral and deep margins. MMS is recommended in cases of aggressive histology, recurrent BCC, and critical areas of skin conservation (head, neck, genitalia, hand/feet, nipples, and so on) and for tumors of large size (more than 2 cm) ^85^.

Electrodessication and curettage (EDC) has been used for decades to treat BCC. Although this can be a cost-effective treatment, cure rates are highly operator-dependent. One study demonstrated five-year recurrence rates at 5.7% to 18.1% depending on the skill level of the physician ^93^. Higher recurrence rates were seen on the forehead, paranasal areas, and nose, and overall cosmesis tends to be poor in the head and neck regions. For these reasons, this treatment is now rarely recommended for BCC in these locations.

### Topical therapy

***Imiquimod.*** Imiquimod 5% is a topical Toll-like receptor 7 agonist approved by the FDA for treatment of superficial BCCs less than 2 cm in diameter. A recent randomized controlled trial comparing imiquimod 5% cream (daily for 6 weeks in superficial BCC and daily for 12 weeks in nodular BCC) with surgical excision found five-year success rates of 82.5% for the imiquimod treatment group and 97.7% for the standard surgical excision group ^94^. A randomized, vehicle-controlled study of subjects with superficial BCC treated with imiquimod 5 to 7 times per week for 6 weeks as compared with vehicle showed composite clinical and histologic clearance rates of 75% for imiquimod 5 times per week and 73% for imiquimod 7 times per week ^95^. Imiquimod is generally well tolerated, but the most common treatment effects include erythema, crusting, erosions, and scabbing, which correlate positively with histologic clearance rates ^96^. A randomized single-blind, non-inferiority randomized controlled trial demonstrated topical treatment with imiquimod 5% (daily, 5 times per week for 6 weeks) to be superior to methylaminolevulinate-photodynamic therapy (MAL-PDT) with the proportion of patients with successful disease clearance at 3 and 12 months to be 72.8% for MAL-PDT as compared with 83.4% for imiquimod ^97^. Imiquimod is considered a useful treatment modality for predominantly superficial BCC in patients who are poor candidates for surgical or destructive modalities.

***5-Fluorouracil.*** 5-Fluorouracil (5-FU) is a topical pyrimidine analog which functions as an antimetabolite, interfering with DNA synthesis. 5-FU is FDA-approved for treatment of superficial BCC. A double-blind randomized trial of 13 patients showed a 90% cure rate in lesions treated with 5% 5-FU twice daily for two weeks ^98^, and another study of 5% 5-FU twice daily for up to 12 weeks showed a similar histologic cure rate of 90%. The efficacy of Efudex 5% was reported to be comparable to that of MAL-PDT and to have a clearance rate of 80.1% in a single-blind, randomized controlled trial ^97^. Erythema, erosions, and ulceration are the most common side effects with use of 5-FU.

***Photodynamic therapy.*** PDT uses photosensitizing agents—aminolevulinic acid (ALA) or MAL—to create a photochemical reaction by producing activated oxygen species that destroy cancer cells when exposed to oxygen and light ^99^. Photosensitizing agents act through intracellular protoporphyrin IX, which preferentially accumulates in tumor tissues. In a randomized prospective study of excision versus MAL-PDT for nodular BCC at five years, the complete response rates were 76% for MAL-PDT and 96% for excisional surgery, and recurrence rates were 14% with MAL-PDT and 4% for those who underwent excision ^100^. A recent randomized controlled trial comparing effectiveness of ALA-PDT with surgical excision found five-year recurrence rates of 30.7% for ALA-PDT and 2.3% for surgical excision ^101^. While recurrence rates are higher for PDT-treated BCCs, cosmesis was found to be superior to surgical excision ^100, 102^. For superficial BCC, complete response rates ranged between 73% and 92%, and some studies suggest improved efficacy with repeated PDT cycles ^97, 103– 105^. Although PDT represents an important treatment modality for those who are not good surgical candidates and have a large burden of superficial disease, surgical interventions should remain the standard of care for invasive disease given high recurrence rates with PDT.

### Radiation therapy

Radiotherapy treatment for BCC can be divided into three main categories: conventional external radiotherapy, superficial x-ray therapy, and brachytherapy. A newer irradiation technique is volumetric modulated arc therapy. This modality allows for complex dose distributions to tumor tissue while minimizing involvement of healthy tissue ^106^. A detailed examination of these modalities is beyond the scope of this review, but radiation is a valuable treatment for non-surgical candidates and patients who decline invasive treatments. Major disadvantages of radiation include multiple visits, lack of confirmation of histologic clearance, development of aggressive phenotypes in some recurrent tumors, poor long-term cosmesis with conventional radiotherapy, and high cost as compared with surgical treatments ^107^. In a randomized trial comparing surgical excision with 2-mm margins versus radiation with brachytherapy, superficial x-ray therapy or conventional radiotherapy showed four-year recurrence rates of 0.7% in patients who underwent surgery versus 7.5% in those who underwent radiotherapy ^108^. Rates of BCC recurrence following superficial x-ray have been reported to be 2% at two years and 4.2% at five years ^109^. Brachytherapy used as treatment for NMSC has been reported to have good cosmesis and recurrence rates of less than 1% to 1.3% after median follow-up times between 4 and 16.1 months ^110^. It is likely that the follow-up interval is not sufficient to determine the true recurrence rates with this modality as compared with more established forms of treatment. External electron beam therapy has shown efficacy rates of 92% at five years in the treatment of NMSC ^111^.

### Chemoprevention

***Nicotinamide.*** Nicotinamide, otherwise known as vitamin B _3_, has emerged as an oral therapy with potential for skin cancer prevention. In one study, nicotinamide was found to reduce UV-induced immunosuppression ^112^. A recent randomized double-blind controlled trial reported a reduction of 20% in new BCCs at 12 months in patients on treatment with 500 mg nicotinamide twice daily ^113^. There are no significant adverse events associated with nicotinamide. While studies have demonstrated a decreased risk of developing NMSC, further investigation is needed to determine the long-term benefits of this treatment ^114^.

### Chemotherapy and immune checkpoint inhibitors

Targeted SMO inhibitors have largely replaced other forms of chemotherapy for advanced or metastatic BCC. Cituximab showed efficacy in certain cases of metastatic BCC or nevoid BCC syndrome ^115, 116^. Newer immunotherapies have emerged that target programmed death 1 (PD-1) immune checkpoint receptors and ligands. A recent case report of a patient with metastatic BCC who failed therapy with vismodegib showed a partial response to an anti–PD-1 monoclonal antibody ^117^. In another recent case report, a 58-year-old male with metastatic BCC who failed therapy with vismodegib, paclitaxel, and cisplatin was placed on the anti–PD-1 antibody, nivolumab, at 240 mg intravenously every 2 weeks. The patient demonstrated near complete resolution of metastatic disease within 4 months of treatment ^118^.

## Conclusions

MMS and surgical excision remain the standard of care for treating BCC. However, new targeted molecular therapies now provide effective treatment options for patients with locally advanced or metastatic BCC. Topical therapies may be effective in treating superficial disease in many cases, although careful surveillance is needed to confirm tumor clearance. Further randomized prospective studies are needed to examine the long-term effectiveness of these alternative modalities and their ability to decrease overall morbidity.
